# Управление безопасностью инкретиновой терапии ожирения: экспертный консенсус по применению семаглутида и тирзепатида

**DOI:** 10.14341/probl13784

**Published:** 2026-07-22

**Authors:** Е. А. Трошина, М. Б. Анциферов, А. С. Аметов, И. Г. Бакулин, Г. Р. Галстян, Т. Н. Маркова, Т. И. Романцова, Н. В. Мазурина, О. М. Котешкова

**Affiliations:** Национальный медицинский исследовательский центр эндокринологии им. акад. И.И. Дедова Россия; Endocrinology Research Centre Russian Federation; Эндокринологический диспансер г. Москвы Россия; Endocrinological Dispensary Russian Federation; Российская медицинская академия непрерывного профессионального образования Россия; Russian Medical Academy of Continuing Professional Education Russian Federation; Санкт-Петербургский государственный университет Россия; St. Petersburg State University Russian Federation; Российский университет медицины Россия; Russian University of Medicine Russian Federation

**Keywords:** ожирение, семаглутид, тирзепатид, безопасность, гастроинтестинальные нежелательные явления, гастроэзофагеальная рефлюксная болезнь, желчнокаменная болезнь, воспалительные заболевания кишечника, микробиота, микронутриенты, obesity, semaglutide, tirzepatide, safety, gastrointestinal adverse events, gastroesophageal reflux disease, gallstone disease, inflammatory bowel disease, microbiota, micronutrients

## Abstract

3 декабря 2025 г. в Москве прошло заседание экспертной рабочей группы, в центре обсуждения которого находились вопросы безопасности и персонифицированного применения тирзепатида («Тирзетта®») и семаглутида («Велгия® эко») у пациентов с избыточной массой тела и ожирением. По итогам заседания была выработана консенсусная позиция по оптимальному применению агонистов инкретиновых рецепторов в реальной клинической практике.

## Abstract (en)

On December 3, 2025, a meeting of the expert working group was held in Moscow. The discussion focused on the safety and personalized use of tirzepatide (Tirzetta®) and semaglutide (Velgia® eco) in patients with overweight and obesity. Following the meeting, a consensus position was developed on the optimal use of incretin receptor agonists in real-world clinical practice.

## Введение

Ожирение представляет собой мультидисциплинарную проблему, достигшую масштабов пандемии, и требует комплексного подхода к лечению, направленного не только на снижение массы тела, но и на долгосрочное управление метаболическим здоровьем пациентов. В последние годы фармакотерапия ожирения совершила прорыв благодаря появлению препаратов, имитирующих действие инкретиновых гормонов. Семаглутид 2,4 мг (Велгия® эко) и тирзепатид (Тирзетта®) зарекомендовали себя как высокоэффективные лекарственные средства, однако вопросы оптимального выбора между ними, а также профилактика и лечение нежелательных явлений (НЯ) в реальной клинической практике остаются предметом активной дискуссии [1].

С целью выработки единых подходов к персонифицированному назначению инкретинов и минимизации рисков НЯ был проведен совет экспертов, объединивший ведущих специалистов в области эндокринологии и гастроэнтерологии. В фокусе обсуждения находились два ключевых аспекта: максимальная эффективность в снижении веса и коррекция связанных с ожирением метаболических нарушений, а также безопасность терапии, прежде всего со стороны желудочно-кишечного тракта (ЖКТ). Эксперты проанализировали современные доказательные данные, включая результаты крупных клинических исследований, систематических обзоров и метаанализов, и на их основе предложили алгоритмы, позволяющие управлять НЯ и учитывать коморбидную патологию.

Настоящая статья обобщает ключевые положения проведенного экспертного совета, представляя практические рекомендации по персонифицированному применению семаглутида и тирзепатида с акцентом на долгосрочное безопасное управление метаболическим здоровьем пациентов.

## Профиль безопасности: управление гастроинтестинальными нежелательными явлениями

Наиболее частыми НЯ, как при применении семаглутида, так и тирзепатида, являются нарушения со стороны ЖКТ. По данным систем фармаконадзора и клинических исследований, частота тошноты, диареи, запоров и рвоты варьирует, но в целом препараты демонстрируют благоприятный профиль безопасности [2][3][4].

В исследовании SURMOUNT-1 частота наиболее распространенных НЯ (тошнота, диарея, запор, рвота) у пациентов, получавших тирзепатид, составила 27,5%, 20,9%, 13,6% и 10,8% соответственно [2]. Систематический обзор и сетевой метаанализ, проведенный H. Yao и соавт. (2024), показал, что тирзепатид демонстрирует сопоставимый с агонистами рецепторов ГПП-1 (арГПП-1) риск НЯ со стороны ЖКТ, однако частота отмены терапии вследствие побочных эффектов была ниже в группе тирзепатида [5]. В исследовании SURMOUNT-5 также подтвержден сходный профиль безопасности семаглутида и тирзепатида у пациентов с ожирением [6].

Важно отметить, что, по данным клинических исследований STEP-1–3, терапия семаглутидом в дозировках 1,0 мг и 2,4 мг имеет сопоставимый профиль безопасности, а частота тошноты в реальной клинической практике не превышает 14% [7][8].

Дополнительные подтверждения сопоставимой переносимости семаглутида и тирзепатида были получены в исследовании SURMOUNT-5 — рандомизированном клиническом исследовании, в котором непосредственно сравнивались эффективность и безопасность семаглутида 2,4 мг и тирзепатида 15 мг у пациентов с ожирением [6]. Результаты продемонстрировали практически идентичную частоту наиболее распространенных гастроинтестинальных НЯ (рис. 1).

**Figure:**
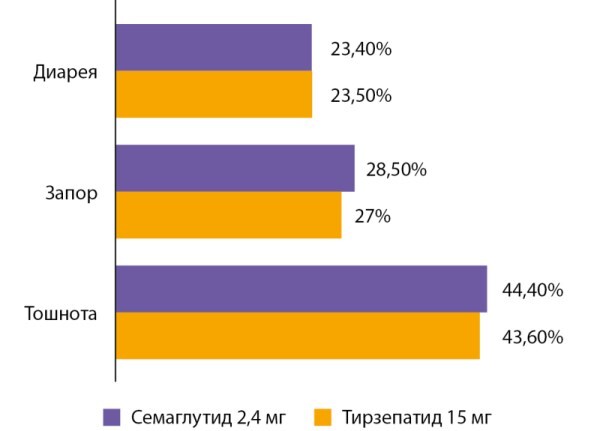
Рисунок 1. Профиль нежелательных явлений семаглутида 2,4 мг и тирзепатида по данным исследования SURMOUNT-5.

Частота тошноты в группе семаглутида составила 44,4%, в группе тирзепатида — 43,6%; диарея наблюдалась у 23,4% и 23,5% пациентов соответственно; запоры — у 28,5% и 27,0% (рис. 1). Общая частота любых НЯ в обеих группах составила 79%. Серьезные НЯ, приведшие к прекращению терапии, зафиксированы у 3,5% участников (18 случаев). Реакции в месте инъекции встречались редко (0,3%). Таким образом, результаты исследования SURMOUNT-5 подтверждают, что семаглутид и тирзепатид обладают сходным профилем безопасности, при этом тирзепатид демонстрирует более высокую эффективность в снижении массы тела [6]. Полученные данные позволяют рассматривать оба фармакоагента как препараты с хорошей переносимостью при условии соблюдения рекомендаций по титрации и управлению гастроинтестинальными симптомами.

## Управление гастроинтестинальными нежелательными явлениями: практические рекомендации

Для минимизации дискомфорта со стороны ЖКТ мультидисциплинарный консенсус экспертов предлагает ряд мер, разделенных на этапы: до начала лечения, на этапе повышения дозы и на этапе поддерживающей терапии [9][10].

## Коррекция пищевых привычек и образа жизни

## Адаптация состава пищи в зависимости от симптомов

## Медикаментозное сопровождение

В терапевтическом арсенале врачей для коррекции и профилактики НЯ со стороны ЖКТ на фоне инкретиновой терапии имеются следующие группы препаратов [10]:

При необходимости данные препараты могут быть назначены «по требованию» для купирования НЯ.

## Ожирение и отдельные заболевания ЖКТ: алгоритмы ведения пациентов на фоне инкретиновой терапии

## Ожирение и гастроэзофагеальная рефлюксная болезнь: патогенетические взаимосвязи и стратегии управления

Ожирение является ведущим модифицируемым фактором риска развития гастроэзофагеальной рефлюксной болезни (ГЭРБ). Метаанализ H. Hampel и соавт. (2005) показал, что избыточная масса тела (ИМТ 25–29,9 кг/м²) повышает риск ГЭРБ в 1,5–2 раза, а ожирение (индекс массы тела (ИМТ)≥30 кг/м²) — в 2–3 раза, причем связь сильнее выражена у женщин [11]. Основной механизм связан с повышением внутрибрюшного давления вследствие накопления висцерального жира, что механически способствует раскрытию нижнего пищеводного сфинктера (НПС) и забросу желудочного содержимого в пищевод.

Абдоминальное ожирение является более точным предиктором риска ГЭРБ, чем ИМТ. В исследовании W.H. Hsu и соавт. (2025) продемонстрировано, что отношение окружности талии к окружности бедер >0,9 у мужчин и >0,85 у женщин независимо ассоциируется с повышенным риском развития эрозивного эзофагита и пищевода Барретта [12]. Кроме того, метаболические нарушения, сопутствующие ожирению, — хроническое системное воспаление (повышение IL-6, TNF-α), инсулинорезистентность и дисбаланс адипокинов (лептин, адипонектин) — могут напрямую влиять на моторику пищевода и висцеральную чувствительность, усугубляя симптомы рефлюкса [13].

Важно подчеркнуть, что снижение массы тела на 5–10% от исходной приводит к статистически значимому уменьшению частоты и интенсивности симптомов ГЭРБ, а также снижению потребности в антисекреторных препаратах [13]. Таким образом, коррекция ожирения является патогенетическим подходом при лечении ГЭРБ.

Применение арГПП-1 и тирзепатида оказывает на течение ГЭРБ двойственный эффект, зависящий от временного горизонта терапии.

В первые недели лечения, особенно в период титрации дозы, возможно временное обострение симптомов ГЭРБ. Ключевой механизм — замедление опорожнения желудка, являющееся одним из основных периферических эффектов инкретинов, способствующих насыщению и снижению веса. Замедленная эвакуация пищи увеличивает внутрижелудочное давление, повышает объем желудочного содержимого и может провоцировать более частые эпизоды рефлюкса, что проявляется изжогой, отрыжкой и чувством тяжести в эпигастрии и переполнения. У пациентов с ГЭРБ в анамнезе в этот период возможно усиление симптомов, что требует предварительного информирования и соответствующей коррекции.

По мере продолжения терапии и достижения устойчивого снижения массы тела, особенно висцерального жира, наблюдается выраженное положительное влияние на течение ГЭРБ. Снижение внутрибрюшного давления и уменьшение механической нагрузки на НПС способствуют значительному улучшению или даже полному исчезновению симптомов рефлюкса. Таким образом, длительная терапия инкретинами может рассматриваться как патогенетическое лечение ГЭРБ, ассоциированной с ожирением.

## Алгоритм ведения пациентов с ГЭРБ на фоне инкретиновой терапии

На основании мультидисциплинарного консенсуса и клинических рекомендаций предложен следующий алгоритм управления пациентами с ГЭРБ при назначении арГПП-1 или тирзепатида (табл. 1) [9][10].

**Table:** Таблица 1. Алгоритм ведения пациентов с ГЭРБ на фоне терапии агонистами ГПП-1 / тирзепатидом

Этап	Действие / Оценка	Решение / Условие	Комментарии / Примечания
1. Предварительная оценка	Оценить степень контроля ГЭРБ перед началом инкретиновой терапии	• Ремиссия / хороший контроль (симптомы отсутствуют или редки, прием ИПП не требуется или минимален) → риск обострения низкий.• Активная ГЭРБ / плохой контроль (ежедневные симптомы, потребность в ИПП, эрозивный эзофагит) → требуется предварительная коррекция	При активной ГЭРБ сначала добиться регресса симптомов (усиление антисекреторной терапии, коррекция образа жизни, исключение триггеров), затем начинать инкретиновую терапию
2. Старт терапии	Начать со стартовой дозы препарата	Следовать стандартным схемам титрации, но с более медленным увеличением (при необходимости продлить фазу титрации на 2–4 недели)	Медленное титрование позволяет ЖКТ адаптироваться и минимизировать обострение симптомов рефлюкса
3. Модификация образа жизни	Дать пациенту четкие рекомендации	• Питание малыми порциями.• Не есть за 3–4 часа до сна.• Исключить триггерные продукты (острое, жирное, кофе, шоколад)	Эти меры снижают внутрижелудочное давление и уменьшают вероятность рефлюкса, особенно в период титрации
4. Мониторинг симптомов	Наблюдать за появлением или усилением изжоги, регургитации, боли в эпигастрии	Симптомы отсутствуют → продолжить титрацию до целевой дозы.Симптомы появились → перейти к этапу 5.	Пациент должен быть информирован о возможности временного ухудшения и о способах его купирования
5. Купирование симптомов	Использовать препараты «по требованию»	• Альгинаты• ИПП• Эзофагопротекторы	Прием «по требованию» позволяет контролировать симптомы без коррекции дозы инкретина
6. Оценка эффективности купирования	Если симптомы сохраняются или усиливаются, несмотря на прием «по требованию»	Перейти на курсовой прием ИПП (например, стандартная доза утром за 30–60 мин до завтрака) и рассмотреть временное снижение дозы инкретина до предыдущего уровня	Временное снижение дозы позволяет ЖКТ адаптироваться; после стабилизации можно вновь попытаться увеличить дозу
7. Рефрактерные симптомы	При сохранении выраженных симптомов, несмотря на курсовой прием ИПП и снижение дозы инкретина	Провести дифференциальную диагностику (исключить эозинофильный эзофагит, другие причины)	Может потребоваться консультация гастроэнтеролога и инструментальное обследование
8. Достижение целевой дозы и снижение веса	После успешной титрации и устойчивого снижения массы тела (обычно через 3–6 мес)	Наступает долгосрочное улучшение ГЭРБ за счет снижения внутрибрюшного давления	Патогенетический эффект, связанный с редукцией висцерального жира
9. Пересмотр терапии ГЭРБ	При стабильном весе и отсутствии симптомов рефлюкса	Рассмотреть возможность снижения дозы или отмены ИПП под контролем симптомов	Полная отмена возможна у части пациентов; у других сохраняется потребность в поддерживающей низкой дозе

## Оценка исходного состояния

Перед началом терапии необходимо оценить степень контроля ГЭРБ:

## Тактика назначения и титрации

## Управление симптомами в период адаптации

## Ожирение и желчнокаменная болезнь: риски и стратегии профилактики на фоне инкретиновой терапии

Ожирение является одним из наиболее значимых модифицируемых факторов риска развития желчнокаменной болезни (табл. 2). Патофизиологической основой служит формирование литогенной желчи — перенасыщенной холестерином, что связано с гиперинсулинемией, инсулинорезистентностью и повышенным синтезом холестерина в печени. Дополнительный вклад вносит гипомоторика желчного пузыря, характерная для пациентов с ожирением. По данным метаанализов, риск развития ЖКБ у лиц с ожирением в 3–4 раза выше по сравнению с лицами с нормальной массой тела [14][15].

**Table:** Таблица 2. Алгоритм ведения пациентов с ЖКБ при назначении инкретиновой терапии

Этап	Действие / Оценка	Решение / Условие	Комментарии / Примечания
1. Предварительная оценка	Провести УЗИ органов брюшной полости до начала терапии. Оценить наличие сладжа, состояние желчного пузыря	• ЖКБ в стадии ремиссии (камни без признаков холецистита) → возможно назначение инкретинов.• ЖКБ в стадии обострения (боли, признаки воспаления) → направить к хирургу, рассмотреть холецистэктомию	При обострении начинать инкретиновую терапию после купирования симптомов и стабилизации
2. Оценка % целевого снижения массы тела	Определить ожидаемый темп снижения массы тела. Планировать темп титрации+	• Высокий риск: ожирение 2–3 ст., ожидаемое снижение веса >15%, темп >1,5 кг/нед., перенесенная бариатрическая операция.• Умеренный риск: избыточная масса тела, ожирение 1 ст., плановая потеря веса 5–15%	Чем выше ожидаемая потеря веса, тем более строгие профилактические меры
3. Медленное титрование	Увеличивать дозу постепенно, по возможности продлевая фазы титрации	Не форсировать достижение максимальной дозы. При необходимости продлить фазу титрации на 2–4 недели	Это позволяет снизить нагрузку на гепатобилиарную систему
4. Диетические рекомендации	Избегать очень низкожировых диет (менее 10–15 г жира в сутки). Обеспечить адекватное потребление жидкости (1,5–2 л/сут)	Умеренное количество полезных жиров (оливковое масло, рыбий жир) стимулирует опорожнение желчного пузыря и предотвращает застой желчи.	Исключить длительное голодание. Прием пищи регулярный, 3–4 раза в день
5. Назначение УДХК для профилактики	При наличии хотя бы одного из критериев:• ИМТ≥35 кг/м² и планируемая потеря веса >15%• Быстрая потеря веса (ожидаемая >1,5 кг/нед.)• Перенесенная бариатрическая операция• Выявленный «сладж» или мелкие камни	Назначить УДХК в дозе 10–15 мг/кг/сут (обычно 500–750 мг/сут) однократно на ночь	Длительность приема: до достижения стабильной массы тела (обычно 6–12 мес). При наличии камней в желчном пузыре вопрос о профилактике решается индивидуально
6. Мониторинг	Повторное УЗИ через 6–12 месяцев или при появлении дискомфорта или болей в правом подреберье, тошноты после жирной пищи — повторное УЗИ	Оценить динамику: появление сладжа, конкрементов, развитие холецистита	При бессимптомном течении продолжение терапии возможно. При развитии холецистита — консультация хирурга

Парадоксальным образом активное снижение массы тела, особенно при темпах более 1,5 кг в неделю, значительно повышает риск образования холестериновых камней. Механизм связан с мобилизацией холестерина из жировой ткани, резким увеличением его экскреции в желчь и снижением сократительной способности желчного пузыря на фоне низкокалорийного питания. Классическое исследование R.L. Gebhard и соавт. (1996) показало, что потеря веса >1,5 кг/нед. увеличивает риск ЖКБ более, чем на 30% [15].

Этот феномен особенно актуален для пациентов, перенесших бариатрические операции, а также для тех, кто достигает выраженного снижения веса на фоне терапии агонистами рецепторов ГПП-1 (арГПП-1) или тирзепатидом. В исследовании SELECT (2024) при применении семаглутида 2,4 мг частота событий со стороны желчного пузыря (холелитиаз, холецистит) составила 2,8% против 1,6% в группе плацебо (риск повышен примерно в 2 раза) [4]. Важно подчеркнуть, что это различие объясняется именно выраженностью потери веса, а не прямым побочным действием препаратов.

## Профилактика ЖКБ: роль урсодезоксихолевой кислоты

Для снижения риска образования камней в условиях быстрой потери веса наиболее изученным и эффективным средством является урсодезоксихолевая кислота (УДХК) [16].

Метаанализ рандомизированных контролируемых исследований продемонстрировал, что на фоне низкокалорийной диеты камни в желчном пузыре сформировались у 23% пациентов без профилактики и только у 5% пациентов, принимавших УДХК. При бариатрических операциях частота образования камней составила 28% в контрольной группе и 9% в группе УДХК соответственно [17].

На основании этих данных американские клинические рекомендации по ведению пациентов после бариатрических операций (2019) предлагают четкие схемы профилактического назначения УДХК [18]:

Схожая тактика ведения больных ожирением с заболеваниями ЖКТ и печени представлена в европейских рекомендациях [19]. Клинические рекомендации, утвержденные Минздравом РФ (2024), также предусматривают назначение УДХК в дозе 10–15 мг/кг/сут пациентам с неалкогольной жировой болезнью печени (НАЖБП) в период снижения массы тела на фоне диетотерапии или бариатрических операций для профилактики образования конкрементов в желчном пузыре до стабилизации массы тела (уровень убедительности A1) [20]. Аналогичная рекомендация включена в клинические рекомендации по ЖКБ (2024) для пациентов после бариатрических вмешательств или соблюдающих низкокалорийную диету (≤800 ккал/сут) с низким содержанием жиров (уровень убедительности B1) [21].

## Воспалительные заболевания кишечника (ВЗК)

Ожирение у пациентов с ВЗК ассоциируется с более агрессивным течением и снижением ответа на терапию [22][23]. Крупное популяционное когортное исследование, проведенное в Израиле с использованием базы данных Epi-IIRN, продемонстрировало, что применение арГПП-1 у пациентов с ВЗК и сопутствующим СД2 или ожирением ассоциируется с улучшением течения заболевания [24]:

Эти данные открывают новые перспективы для применения инкретинов у данной категории пациентов, однако требуют дальнейшего изучения.

## Модуляция микробиоты кишечника

Агонисты ГПП-1 оказывают мощное модулирующее действие на микробиом кишечника. Наблюдается увеличение разнообразия микробиоты, снижение числа провоспалительных бактерий (Enterobacteriaceae, Desulfovibrionaceae) и увеличение численности полезных комменсалов, таких как Akkermansia muciniphila (укрепляющая слизистый барьер), и бактерий, продуцирующих короткоцепочечные жирные кислоты (Faecalibacterium prausnitzii, Roseburia spp.), что способствует улучшению чувствительности к инсулину и снижению системного воспаления [25]. Эти изменения коррелируют с клиническими исходами: более значительным снижением массы тела, улучшением гликемического контроля и липидного профиля.

## Витаминный и элементный статус

Значительное снижение массы тела, достигаемое на фоне терапии инкретинами, может сопровождаться риском дефицита микронутриентов, что хорошо известно по опыту бариатрической хирургии и низкокалорийных диет [18][26]. Наиболее частым является дефицит витамина D, затем витаминов A, E, K, B12, фолиевой кислоты, а также железа, меди, цинка, магния и кальция. Пациентам, особенно с симптомами со стороны ЖКТ или низким потреблением пищи, рекомендуется рассмотреть ежедневный прием поливитаминов и регулярный мониторинг микронутриентного статуса.

## Заключение

Семаглутид 2,4 мг (Велгия ЭКО®) и тирзепатид (Тирзетта®) представляют собой высокоэффективные классы препаратов для лечения ожирения. Персонифицированный подход, учитывающий степень ожирения, цели по снижению веса, наличие сердечно-сосудистых заболеваний и предыдущий опыт терапии позволяют оптимизировать клинические результаты. Профиль безопасности обоих препаратов является благоприятным, а гастроинтестинальные НЯ в большинстве случаев управляемы с помощью коррекции диеты, изменения образа жизни и рациональной фармакотерапии. Глубокое понимание взаимодействия инкретинов с ЖКТ, включая их влияние на ГЭРБ, ЖКБ, ВЗК и микробиоту, позволяет врачам различных специальностей (эндокринологам, терапевтам, гастроэнтерологам, кардиологам) эффективно и безопасно применять эти препараты в клинической практике для управления метаболическим здоровьем и контроля сопутствующих заболеваний, вызванных избытком жировой ткани.

## Дополнительная информация

Источники финансирования. Работа выполнена по инициативе авторов без привлечения финансирования.

Конфликт интересов. Авторы декларируют отсутствие конфликта интересов.

Участие авторов. Все авторы одобрили финальную версию статьи перед публикацией, выразили согласие нести ответственность за все аспекты работы, подразумевающую надлежащее изучение и решение вопросов, связанных с точностью или добросовестностью любой части работы.
